# Computed tomographic evaluation of canine hepatic lymph nodes: Establishment of aorta-normalized dimension ratio cutoff values for differentiating normal from abnormal conditions

**DOI:** 10.14202/vetworld.2026.840-849

**Published:** 2026-02-28

**Authors:** Yannawit Hirunyasuwan, Pongpol Chaiyatadsakun, Ussana Hittrawat, Patthana Wanicharat, Chutimon Thanaboonnipat, Somchin Sutthigran, Nan Choisunirachon

**Affiliations:** Department of Surgery, Faculty of Veterinary Science, Chulalongkorn University, Bangkok 10330, Thailand

**Keywords:** abdominal pathology, aorta ratio, canine, computed tomography, cutoff value, hepatic lymph node, lymphadenopathy

## Abstract

**Background and Aim:**

Hepatic lymph nodes (HLNs) drain lymph from the liver, gallbladder, pancreas, stomach, and duodenum. Enlargement of these nodes may indicate pathology in those organs, but objective, breed-independent computed tomography (CT)-based size criteria have not been established. This study aimed to determine quantitative cutoff values for HLN dimensions normalized to aortic diameter (Ao) to differentiate normal from abnormal HLNs in dogs.

**Materials and Methods:**

This retrospective study analyzed contrast-enhanced abdominal CT scans (slice thickness <1.5 mm) from 84 dogs older than 1 year, examined between March 2016 and November 2022. Dogs were divided into two groups: no targeted organ abnormalities (26 dogs; 46 HLNs) or presence of targeted organ abnormalities in the liver, gallbladder, pancreas, stomach, duodenum, or combinations (58 dogs; 89 HLNs), based on clinical, laboratory, and imaging findings. Using multiplanar reconstruction, the maximum length (cranio-caudal), width (latero-lateral), and height (dorso-ventral) of each HLN were measured and divided by the Ao measured at the same level. Parameters were compared between groups using Mann–Whitney U tests. Receiver operating characteristic curves were used to identify optimal cutoff values, sensitivity, specificity, and area under the curve (AUC).

**Results:**

HLN dimensions and dimension-to-Ao ratios were significantly larger in dogs with targeted organ abnormalities (all p < 0.001). Mean width-to-Ao ratio, length-to-Ao ratio, and height-to-Ao ratio were 0.7 ± 0.3, 1.4 ± 0.7, and 0.6 ± 0.3 in the normal group versus 1.2 ± 1.2, 2.0 ± 0.8, and 0.8 ± 0.4 in the abnormal group, respectively. The width-to-Ao ratio showed the highest diagnostic performance (AUC = 0.76, 95% confidence interval: 0.69–0.85; p < 0.0001), with an optimal cutoff of ≥ 0.75 (sensitivity 70%, specificity 67%). Height-to-Ao ratio cutoff was ≥ 0.615 (AUC = 0.70), and length-to-Ao ratio cutoff was ≥ 1.58 (AUC = 0.70).

**Conclusion:**

Ao-normalized HLN dimension ratios provide an objective, breed- and body-size-independent criterion for CT evaluation of canine hepatic lymph nodes. A width-to-Ao ratio ≥ 0.75 offers the best balance of sensitivity and specificity for identifying HLN abnormalities suggestive of targeted organ pathology. These cutoff values support non-invasive CT-based assessment but should be interpreted with clinical and laboratory findings; histopathology remains essential for definitive diagnosis.

## INTRODUCTION

Hepatic lymph nodes (HLNs) are categorized as visceral abdominal lymph nodes [1]. They consist of right and left sides, located on each side of the portal vein near the liver root. These lymph nodes play an important role in filtering lymph fluid and eliminating waste products, pathogenic organisms, or foreign substances from the body, particularly from targeted organs such as the hepatobiliary system, stomach, pancreas, and duodenum, including surrounding structures [1, 2].

Normal HLNs may vary in size, number, and shape (e.g., round or elongated) [3]. A previous study found that 66.3% of HLNs were elongated, while others had different shapes or were absent [3]. The normal length of canine HLNs ranges from 4.50 to 59.7 mm [3]. The left hepatic lymph node is located dorsal to the common bile duct in the lesser omentum and is longer and larger than the right hepatic lymph node [4]. The right hepatic lymph node may consist of 1–5 individual nodes that vary in size and shape [1].

In dogs, hepatic lymph node enlargement may result from various conditions, including infection, inflammation, neoplasia, immune-mediated disorders, hematological abnormalities, and immunological responses. Clinical assessment of lymph nodes and adjacent structures is essential to identify the underlying disease and for disease staging. Several medical imaging modalities, such as radiography, ultrasonography (US), and computed tomography (CT), can be used to visualize the anatomical structure of HLNs.

Radiography is a quick, inexpensive modality with straightforward interpretation. However, abdominal radiography has low contrast resolution, particularly in the cranial abdomen, making it difficult to differentiate small cranial abdominal soft tissue structures, such as the gallbladder, pancreas, adrenal glands, and lymph nodes, from one another [5].

In addition to radiography, US is a radiation-free imaging modality that effectively differentiates soft tissues. It can reveal the size, shape, and location of organs in real-time and provides a non-invasive method for soft tissue assessment [6]. However, US can be challenging for evaluating intra-abdominal visceral lymph nodes, especially in patients with substantial gastrointestinal gas or food content that creates masking artifacts over the lymph nodes [7, 8].

In contrast, CT provides volumetric data [9, 10] that can be displayed in multiple planes, thereby reducing superimposition effects. It offers detailed anatomical information about small structures that previous imaging methods, such as radiography and US, could not adequately achieve [9–11].

Previous studies have used CT to evaluate normal canine abdominal lymph nodes [3]. Because body weight (BW) in dogs varies due to breed-related phenotypic differences [3], the dimensions of normal lymph nodes consequently vary among dogs of different sizes. In addition to BW, age is considered a parameter of normalcy [3]. A previous study demonstrated a relationship between organ dimensions and aortic luminal diameter (Ao), as observed for the prostate gland [12] and medial iliac lymph nodes [13], thereby eliminating the influence of body size on dimension assessment of canine anatomical structures. Dimensions such as width, length, and height of the medial iliac lymph nodes were linearly correlated with the Ao diameter [13]. Larger animals tend to have larger Ao and medial iliac lymph nodes. Therefore, the ratio between medial iliac lymph node dimensions and Ao can be used to assess lymph node size across all dog breeds, and this measurement is independent of BW [13].

Therefore, this retrospective study aimed to measure HLN dimensions on contrast-enhanced CT images, normalize them to Ao, and compare these ratios between dogs with and without abnormalities of the targeted drainage organs (liver, gallbladder, pancreas, stomach, and duodenum). We hypothesized that HLN dimension-to-Ao ratios would differ significantly between normal and abnormal groups, enabling the establishment of clinically applicable cutoff values that provide the first objective, breed- and body size-independent CT criterion for assessing canine HLN pathology.

## MATERIALS AND METHODS

### Ethical approval

The study protocol was reviewed and approved by the Institutional Animal Care and Use Committee of the Faculty of Veterinary Science, Chulalongkorn University, under protocol number S447/2566 dated 13-09-2023. All procedures adhered to the institutional animal care guidelines and the ethical principles outlined in the “Guide for the Care and Use of Laboratory Animals” (National Research Council, 2011) and the Animal Research: Reporting of *In Vivo* Experiments 2.0 guidelines. Informed consent for the use of clinical and imaging data for research purposes was obtained from the owners at the time of each dog’s registration and hospital admission. No additional invasive procedures were performed specifically for this retrospective study, as all data were collected during routine clinical diagnostic processes. Owners were informed that their dog’s anonymized data could be used for research, and they had the right to withdraw consent at any time by contacting the hospital administration. The study involved no harm or additional risk to the animals, as it was based solely on existing medical records and archived imaging data.

### Study design, period, and location

This retrospective study utilized data from dogs that underwent diagnostic CT scans at the Diagnostic Imaging Unit, The Small Animal Hospital, Faculty of Veterinary Science, Chulalongkorn University, Bangkok, Thailand, between March 2016 and November 2022. Clinical demographic data were extracted from the hospital information system, and corresponding CT images were retrieved from the Picture Archiving and Communication System. All analyses were performed on de-identified data to ensure confidentiality.

### Case selection criteria

Clinical demographic and CT image data were screened and collected. Inclusion criteria were as follows: canine patients aged >1 year with complete clinical demographic data (breed, sex, BW, and complete blood profiles and blood chemistry results within one week before CT acquisition) and abdominal contrast-enhanced CT data with slice thickness <1.5 mm. Exclusion criteria included incomplete clinical demographic data or incomplete/poor-quality abdominal CT images. Eligible cases were categorized into two groups: dogs without targeted organ abnormalities (no lesions in the liver, gallbladder, pancreas, stomach, or duodenum based on normal laboratory and diagnostic imaging results) and dogs with targeted organ abnormalities (lesions in these organs detected on laboratory or diagnostic imaging results).

### Image analysis and measurement

All contrast-enhanced CT images were acquired using a 64-slice scanner (Optima 660, General Electric, Tokyo, Japan) and stored in Digital Imaging and Communications in Medicine format. Images were reanalyzed using Horos project version 3.3.6 (The Horos Project, Annapolis, MD, USA) with a soft tissue window (window width 350 HU, window level 50 HU).

**Figure 1:**
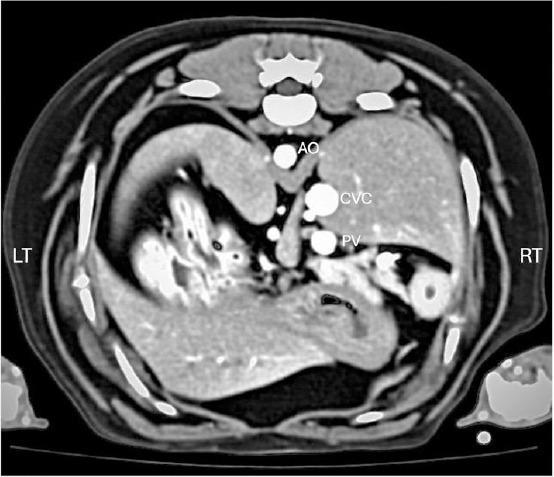
Post-contrast transverse computed tomographic image of the canine cranial abdomen demonstrating the location of the hepatic lymph nodes (arrowheads) relative to major intra-abdominal vessels: aorta (Ao), caudal vena cava (CVC), and portal vein (PV).

Multiplanar reconstruction (MPR) was initially applied to optimize abdominal cavity positioning and clearly display hepatic lymph nodes (HLNs) as unique craniocaudal, oval-to-elliptical structures near the portal vein (PV) at the porta hepatis, along with major intra-abdominal vessels including Ao and caudal vena cava (CVC) (Figure 1).

**Figure 2:**
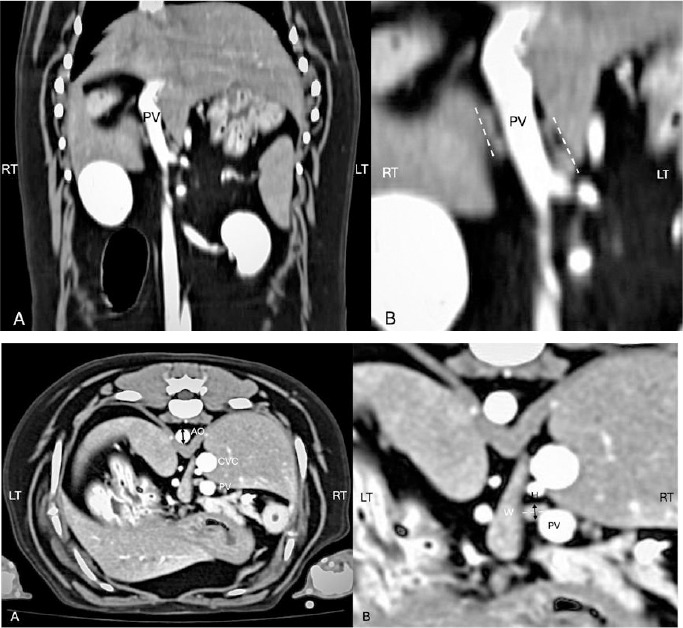
(A) Post-contrast dorsal computed tomographic image of the canine cranial abdomen showing the hepatic lymph node and portal vein (PV). (B) Magnified view of (A) illustrating the measurement of hepatic lymph node length (L) along the craniocaudal direction.

**Figure 3:**
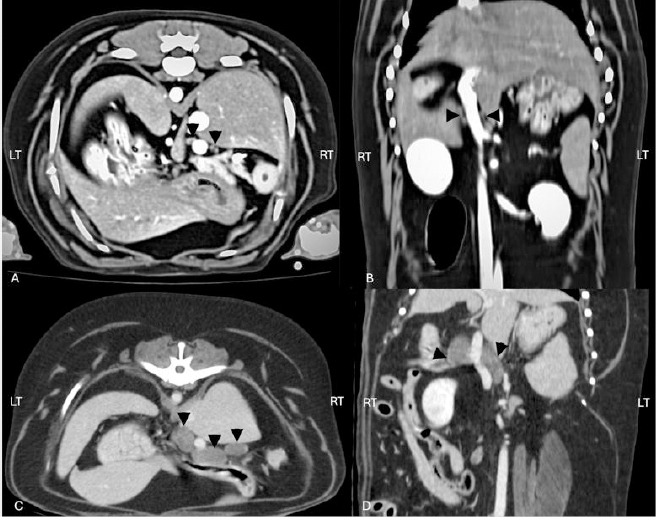
(A) Post-contrast transverse computed tomographic image of the canine craniodorsal abdomen demonstrating the method for measuring aortic diameter (Ao; black arrow). (B) Magnified view showing measurement of hepatic lymph node width (W; white dashed line) in the latero-lateral direction and height (H; black arrow) in the dorso-ventral direction.

CT evaluation was performed in random order. The number and characteristics of HLNs were first recorded. MPR was then adjusted to enhance HLN visualization. Width, length, and height of HLNs on both sides were blindly measured using a digital caliper by one of four well-trained authors (YH, PC, UH, and PW). All images were reviewed for qualification by a Thai board-certified veterinary radiologist (N. C.). Each dimension was measured three times in millimeters (mm) and averaged. Length was defined as the maximum craniocaudal dimension on the dorsal plane (Figure 2). Width and height were measured in latero-lateral and dorso-ventral axes, respectively, perpendicular to length on the transverse plane (Figure 3). The Ao diameter was measured edge-to-edge at the same transverse plane as the HLNs (Figure 3). HLN dimension-to-Ao ratios were calculated as [lymph node dimension (mm) / Ao diameter (mm)].

### Statistical analysis

All statistical analyses were performed using GraphPad Prism version 10.0a (GraphPad Software, San Diego, CA, USA). Detectable lymph node measurements were pooled, averaged, and normalized to Ao for consistency. Normality of parameters (including lymph node dimensions and dimension-to-Ao ratios) was assessed within the 95th percentile interval using the Shapiro–Wilk test. Descriptive statistics were reported as mean, median, range, and standard deviation. Comparisons of dimension-to-Ao ratios between dogs with and without targeted organ abnormalities were performed using a two-tailed Mann–Whitney U test. Receiver operating characteristic (ROC) curves were constructed to evaluate predictive performance, including area under the curve (AUC) with 95% confidence interval (95% CI) for sensitivity and specificity. Optimal cutoff values were determined using the Youden index. AUC interpretation followed standard guidelines: 0.5 indicates no discrimination, 0.7–0.8 acceptable, 0.8–0.9 excellent, and >0.9 outstanding [14]. A p-value <0.05 was considered statistically significant.

## RESULTS

### Demographic data

Eighty-four dogs met the inclusion criteria. Of these, 26 (31%) were assigned to the group without targeted organ abnormalities, and 58 (69%) were assigned to the group with targeted organ abnormalities. Among dogs with targeted organ abnormalities, 30 (51.7%) had lesions localized to the liver and gallbladder, 3 (5.2%) had pancreatic lesions, 10 (17.2%) had gastrointestinal lesions, and 15 (25.9%) had combination lesions involving multiple organs.

The mean age of all dogs was 9.0 ± 4.1 years (range 1.0–16.0 years), and the mean BW was 16.8 ± 11.3 kg (range 2.9–47.5 kg). There were 41 (48%) male dogs and 43 (52%) female dogs. No significant differences were found in age or BW between the groups. The mean age was 8.3 ± 4.7 years in dogs without targeted organ abnormalities versus 9.2 ± 3.8 years in dogs with targeted organ abnormalities (*p* = 0.55). The mean BW was 14.3 ± 11.1 kg versus 19.0 ± 13.8 kg, respectively (*p* = 0.10).

Breed distribution included 20 (23%) mixed-breed dogs, 8 (9.5%) Shih Tzus, and 56 (66.6%) dogs of other breeds (Poodle, Pekingese, Pug, French Bulldog, Miniature Pinscher, Miniature Schnauzer, Beagle, Thai, Siberian Husky, Tibetan, Yorkshire Terrier, Rottweiler, English Cocker Spaniel, Bangkaew, Golden Retriever, American Pitbull, German Shepherd, Finnish Spitz, Chihuahua, Pomeranian, Welsh Corgi, Labrador Retriever, and Dutch Smoushond). The mean Ao was 8.3 ± 2.3 mm in dogs without targeted organ abnormalities and 8.7 ± 2.3 mm in dogs with targeted organ abnormalities, with no significant difference between groups (p = 0.49).

### Hepatic lymph node characteristics

A total of 135 HLNs were evaluated from both groups. There were 46 HLNs from 26 dogs without targeted organ abnormalities and 89 HLNs from 58 dogs with targeted organ abnormalities. In the group without abnormalities, 5 dogs (19.2%) had only one HLN site visible, while 21 dogs (80.8%) had both right and left HLN sites. Two dogs showed only the right HLN, and 4 dogs showed only the left HLN. In the group with abnormalities, 26 dogs (44.8%) had only one HLN site, while 32 dogs (55.2%) had both sites. Eight dogs showed only the right HLN, and 19 dogs showed only the left HLN.

### Hepatic lymph node dimensions and ratios

In dogs without targeted organ abnormalities, the mean HLN width, length, and height were 5.7 ± 3.0 mm (range 1.8–18.8 mm), 12.2 ± 7.3 mm (range 3.6–37.6 mm), and 5.0 ± 3.2 mm (range 1.8–19.1 mm), respectively. The corresponding mean ratios were width-to-Ao 0.7 ± 0.3 (range 0.2–2.3), length-to-Ao 1.4 ± 0.7 (range 0.5–3.1), and height-to-Ao 0.6 ± 0.3 (range 0.2–1.6).

In dogs with targeted organ abnormalities, the mean HLN width, length, and height were 10.1 ± 8.2 mm (range 1.5–68.2 mm), 17.2 ± 8.9 mm (range 3.9–51.5 mm), and 7.5 ± 4.5 mm (range 1.6–26.1 mm), respectively. The corresponding mean ratios were width-to-Ao 1.2 ± 1.2 (range 0.2–11.1), length-to-Ao 2.0 ± 0.8 (range 0.6–4.2), and height-to-Ao 0.8 ± 0.4 (range 0.3–2.2).

**Figure 4:**
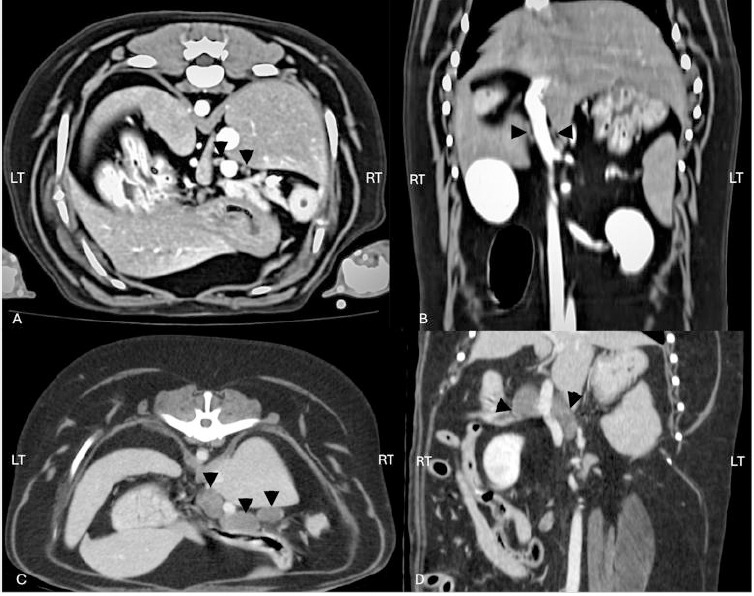
(A) Post-contrast transverse computed tomographic image of the canine cranial abdomen in a dog without targeted organ abnormalities, showing hepatic lymph nodes (arrowheads) of normal size and appearance. (B) Post-contrast dorsal computed tomographic image of the same dog as in (A), illustrating the normal hepatic lymph nodes (arrowheads) in the craniocaudal plane. (C) Post-contrast transverse computed tomographic image of the canine cranial abdomen in a dog with targeted organ abnormalities, demonstrating enlarged hepatic lymph nodes (arrowheads) with increased dimensions. (D) Post-contrast dorsal computed tomographic image of the same dog as in (C), showing the enlarged hepatic lymph nodes (arrowheads) and their altered morphology compared to the normal group.

All measurements and derived ratios were compared between groups as detailed in Table 1 and illustrated in Figure 4.

**Table 1:** Hepatic lymph node dimensions (mm) and dimension-to-aortic diameter ratios in dogs with and without targeted organ abnormalities.

Parameter	Normal (n = 46)	Abnormal (n = 89)	p-value
Width (mm)	5.7 ± 3.0 4.9 (1.8–18.8)	10.1 ± 8.2 7.8 (1.5–68.2)	< 0.0001
Width/Ao	0.7 ± 0.3 0.6 (0.2–2.3)	1.2 ± 1.2 0.9 (0.2–11.1)	< 0.0001
Length (mm)	12.2 ± 7.3 9.5 (3.6–37.6)	17.2 ± 8.9 15.7 (3.9–51.5)	0.0006
Length/Ao	1.4 ± 0.7 1.3 (0.5–3.1)	2.0 ± 0.8 1.9 (0.6–4.2)	0.0003
Height (mm)	5.0 ± 3.2 4.4 (1.8–19.1)	7.5 ± 4.5 5.9 (1.6–26.1)	0.0002
Height/Ao	0.6 ± 0.3 0.5 (0.2–1.6)	0.8 ± 0.4 0.7 (0.3–2.2)	< 0.0001

Data are presented as mean ± standard deviation, median (range). Comparisons between groups were performed using the Mann–Whitney U test.

### Diagnostic performance of cutoff values

Cutoff values for the mean HLN width-to-Ao ratio, length-to-Ao ratio, and height-to-Ao ratio to differentiate dogs with and without targeted organ abnormalities, including sensitivity (%), specificity (%), 95% CI, and AUC, are presented in Table-2. The width-to-Ao ratio showed the highest diagnostic accuracy, followed by the height-to-Ao ratio, while the length-to-Ao ratio demonstrated less pronounced but still significant discriminatory ability between groups.

**Table-2:** Cutoff values, sensitivity, specificity, and area under the curve for hepatic lymph node dimension-to-aortic diameter ratios in differentiating dogs with and without targeted organ abnormalities.

Parameter	Cutoff value	Sensitivity (%) (95% CI)	Specificity (%) (95% CI)	AUC (95% CI)	p-value
Width/Ao	0.750	70 (59.46–78.24)	67 (52.97–79.13)	0.76 (0.69–0.85)	<0.0001
Length/Ao	1.58	65 (54.83–74.25)	63 (48.60–75.48)	0.70 (0.59–0.78)	0.0004
Height/Ao	0.615	69 (59.46–78.24)	63 (48.60–75.48)	0.70 (0.61–0.80)	<0.0001

AUC = Area under the curve, CI = Confidence interval. Optimal cutoffs were determined using the Youden index from receiver operating characteristic analysis.

## DISCUSSION

### Novelty and clinical significance

To the best of our knowledge, this is the first study to establish quantitative cutoff ratios for HLN dimensions normalized to Ao in dogs. These ratios provide an objective, breed-independent criterion that can enhance CT-based diagnosis of hepatic and adjacent organ pathologies.

### Physiological role of HLNs

Lymph from the liver, gallbladder, stomach, pancreas, duodenum, and surrounding organs is drained into HLNs [1, 2]. Within HLNs, the lymph fluid is filtered, foreign compounds, including pathogens, are detected and targeted, and immune cells are activated [15]. After processing in HLNs, the lymph fluid continues to flow through the lymphatic system, eventually draining into larger lymphatic vessels and returning to the circulation [4].

### Hepatic lymphadenopathy as a diagnostic indicator

Hepatic lymphadenopathy is an important indicator of hepatic disorders or diseases affecting the gallbladder, stomach, pancreas, and duodenum [2]. Although radiographs may occasionally be utilized, their diagnostic value is limited due to the superimposition of organ silhouettes, which results in insufficient detail for clear visualization of HLNs [16].

### Limitations of ultrasonography

US is a widely accessible imaging modality that provides real-time visualization of HLNs and surrounding structures, making it a commonly used assessment technique [17]. Its non-invasive nature and ability to capture dynamic images contribute to its practical application in clinical settings. However, several factors can influence the effectiveness of US, including operator dependency, interference from gastrointestinal gas, and reduced efficacy in animals with excessive body fat [6]. Additionally, the depth and location of lymph nodes within the abdominal cavity, as well as the presence of gas in adjacent organs, may affect image quality [2, 6].

### Advantages of CT

In contrast, CT provides several significant advantages. CT offers three-dimensional cross-sectional imaging, eliminating the superimposition of organs seen on radiographs and enabling clearer visualization of both normal and abnormal lymph nodes [3, 18–21]. In addition, CT excels in identifying small or deep-seated lesions that may not be detectable using US [9, 10, 21]. Furthermore, the consistent image quality of CT reduces operator dependency, making it a more reliable modality and potentially enabling further development of AI-based evaluation. Contrast-enhanced CT images enable better delineation of lymph nodes and surrounding tissues and facilitate vascularization assessment, which can help distinguish between inflammatory and neoplastic processes [19, 22, 23]. The combination of these features allows for a more precise evaluation of HLNs and their potential systemic implications [24]. Moreover, the ability of CT to evaluate adjacent organs, such as the pancreas, duodenum, and gallbladder, provides a broader diagnostic scope, which is crucial for complex or multi-organ diseases. Given these advantages, contrast-enhanced CT is recommended as the preferred modality for detailed and comprehensive assessment of hepatic lymphadenopathy, especially in cases where US findings are inconclusive or radiographs are insufficient [25, 26].

### Study population and lesion patterns

This study collected data from 26 dogs without and 58 dogs with targeted organ abnormalities, with variations in BW, age, and breed. The results did not reveal any significant relationship between BW, age, breed, and HLN abnormalities. The age range of both groups, spanning from 1 to 16 years, highlighted the diversity of underlying conditions, including infectious, neoplastic, and degenerative diseases.

Further categorization of cases in dogs with targeted organ abnormalities revealed specific patterns of lesions associated with HLN abnormalities. Most dogs (n = 30, 51.7%) had lesions localized at the liver and gallbladder. Pancreatic lesions were less common, identified in 3 dogs (5.2%). Gastrointestinal lesions were observed in 10 dogs (17.2%). Additionally, 15 (25.9%) dogs presented with a combination of lesions involving more than one organ system, including the liver, gallbladder, pancreas, or gastrointestinal tract.

### Anatomical considerations and bilateral drainage

The drainage of both the left and right HLNs is common in some areas, with both nodes receiving lymphatic drainage from one another [1, 2]. This anatomical characteristic explains why significant changes can be observed in HLNs of the diseased groups when values from both sides are analyzed. However, evidence and research on the specific changes occurring in distinct sites of HLNs related to diseased afferent drainage organs are still lacking. Further studies are needed in this area.

### Influence of intra-abdominal fat on lymph node visibility

Although the comparison of lymph node detection among dogs with different degrees of intraperitoneal fat accumulation was not performed in the current study, the number of detected lymph nodes would be affected by intra-abdominal fat accumulation surrounding the HLNs. A previous study reported that lymph nodes were visible in dogs with more intra-abdominal fat, as differentiating the lymph node from adjacent soft tissue structures is easier due to its surrounding fat [3, 25]. In addition, dogs with any disorders of the body that can alter metabolism and expenditure may influence the amount of subcutaneous and intra-abdominal fat [27]. Therefore, dogs with reduced intra-abdominal fat may exhibit lower lymph node visibility. This study revealed that both dogs with and without targeted organ abnormalities can display only one side of the HLN on CT images.

### Value of aorta-normalized ratios

Variations in size and BW among dog breeds, driven by breed-specific phenotypic characteristics [3], could lead to differences in the size of lymph nodes in dogs without targeted organ abnormalities. A previous study has elucidated a relationship between the Ao diameter and medial iliac lymph nodes. The Ao serves as a reliable index for determining medial iliac lymph node size, unaffected by breed or size differences [13]. Similar to the medial iliac lymph node, the normalized value of the lymph node dimensions-to-Ao ratio can minimize variation in body size and BW from breed differences and can be applied as clinically supporting information to differentiate lymph nodes with and without targeted organ abnormalities. Further study for other node sites would elucidate the benefit of Ao-normalized lymph node cutoff ratios for clinical diagnosis.

### Diagnostic performance of dimension ratios

According to the results, the HLN width-to-Ao ratio has the highest sensitivity and specificity compared with the HLN length-to-Ao ratio and the HLN height-to-Ao ratio. Compared with the length and height, the width is rapidly measurable and diagnostically practical because of its prominent structure and its distinctness from the PV and other blood vessels observed on the transverse image. Nevertheless, the HLN dimension may be affected by the caliper placement and oblique position of lymph nodes in the craniocaudal direction on MPR imaging. The craniocaudal dimension was estimated based on the thickness of all transverse slices that contained a part of the lymph node, and the lymph nodes may not be present at the full height of the most cranial and caudal slices; thus, this dimension may have been over- or underestimated [25]. In addition to the single dimension, a previous study revealed that an oval-shaped lymph node with a short/long axis ratio > 0.5, defined as rounded, was indicative of malignancy [25]. Despite the highest sensitivity and specificity of the HLN width-to-Ao ratio compared with height and length, this criterion is suitable for clinically detecting HLN abnormalities, but it cannot replace lymph node biopsy, which can reveal the actual cause of lymphadenopathy. In addition, the current study revealed differences only between the HLN dimension-to-Ao ratio of dogs with and without targeted organ abnormalities. Among dogs with targeted organ abnormalities, information can overlap between benign reactive and malignant processes. A prospective study is needed to explore more information.

### Limitations and future directions

The vulnerability of the results may impact the ROC curve, resulting in variability in severity categorization. The type and severity of disease were not stratified in relation to the lesion of the node, which could be characterized by individual attenuation of the lymph nodes or confirmed through histopathological findings. Differences in outcomes may arise between mild and severe lesions, which warrants further investigation. Differences in lymph node attenuation between dogs without or with targeted organ abnormalities have been described in human literature but have not been investigated in dogs. Severe lymph node enhancement was described in a dog with granulomatous lymphadenitis of a tracheobronchial lymph node, but no HU values were reported [25]. The lymph node homogeneity may also present abnormality [3]. Heterogeneous, “ring” or absent enhancement of lymph nodes in CT examination has been described in veterinary and human medicine, usually as an expression of pathological processes such as inflammation or metastasis [3]. Nevertheless, this study was not concerned with the number of lymph nodes attenuated because of the variation in the time of post-contrast scanning, and the contrast enhancement may interfere with the lymph node attenuation value. Furthermore, post-contrast scans could be delayed in some patients in whom the abdomen was followed by another interesting region.

Due to the retrospective nature of the current study, data distribution between dogs’ breed, distribution groups of dogs, and the distribution of dogs in each targeted organ abnormality are difficult to set and may contribute to potential selection bias. In addition, non-standardized CT protocols and intra- and inter-observer reliability were not performed in the current study. Further prospective study with a large sample size not only for dogs but also for other species would be more useful in elucidating the benefit of Ao-normalized lymph node cutoff ratios. Some data of all samples, such as body condition scores (BCS) on the same day of CT scan and some blood chemistry values of hepatic function, such as ammonia, bile acid, and bilirubin, including hydration status, were not collected in some dogs due to the retrospective study design, and these parameters would cause the discrepancy of the results. The BCS of each individual dog could decrease the bias of BW and may encourage the correlation between intra-abdominal fat and abdominal lymph node visibility.

Without histopathology, definitively characterizing lesions in HLNs is challenging. Key details, such as the type, extent, and severity of pathological changes, cannot be determined. HLNs are located deep within the abdominal cavity, posing significant challenges for sample collection. In some cases, laparotomy may be required for biopsy. These limitations emphasize the importance of histopathological analysis in providing a definitive diagnosis and thorough assessment.

## CONCLUSION

This retrospective CT study of 135 HLNs from 84 dogs established Ao-normalized dimension ratios to differentiate normal from abnormal HLNs. HLN dimensions and ratios were significantly larger in dogs with targeted organ abnormalities (all p < 0.001). Mean width-to-Ao, length-to-Ao, and height-to-Ao ratios were 0.7 ± 0.3, 1.4 ± 0.7, and 0.6 ± 0.3 in the normal group versus 1.2 ± 1.2, 2.0 ± 0.8, and 0.8 ± 0.4 in the abnormal group. ROC analysis identified optimal cutoffs: width-to-Ao ≥ 0.75 (AUC 0.76, sensitivity 70%, specificity 67%), height-to-Ao ≥ 0.615 (AUC 0.70), and length-to-Ao ≥ 1.58 (AUC 0.70). The width-to-Ao ratio demonstrated the highest diagnostic accuracy.

These Ao-normalized HLN ratios offer a breed- and BW-independent tool for non-invasive CT assessment of canine abdominal pathology, aiding early detection of lesions in the liver, gallbladder, pancreas, stomach, or duodenum. Clinicians can integrate these cutoffs into routine imaging protocols to improve disease staging, differentiate reactive from neoplastic changes, and guide biopsy decisions, potentially reducing unnecessary invasive procedures while enhancing diagnostic confidence in diverse canine populations.

The study’s strengths include a sizable cohort (84 dogs, 135 HLNs) with diverse breeds, ages, and BW, ensuring broad applicability. Use of contrast-enhanced CT with MPR for precise measurements, blinded assessments by trained evaluators, and statistical rigor (e.g., Mann–Whitney U tests, ROC curves) minimized bias. Normalization to Ao eliminated size variability, providing the first quantitative, objective cutoffs for canine HLNs, surpassing prior absolute measurement studies.

As a retrospective design, potential selection bias arose from non-randomized grouping and uneven lesion distribution (e.g., 51.7% liver/gallbladder-focused). Lack of histopathology limited differentiation between benign and malignant abnormalities, and unassessed factors like intra-abdominal fat, BCS, hydration status, or post-contrast timing may have influenced visibility and HU values. Non-standardized CT protocols and absence of inter-observer reliability testing further constrain generalizability.

Prospective studies with larger, multi-species cohorts should validate these cutoffs, stratify by lesion severity/type, and incorporate histopathology for precise etiology correlation. Exploring AI-assisted CT analysis, attenuation patterns (e.g., heterogeneous enhancement), and site-specific HLN changes (left vs. right) could refine diagnostics. Extending Ao-normalization to other lymph nodes and integrating BCS or metabolic markers may address fat-related visibility biases.

In summary, Ao-normalized HLN dimension ratios, particularly width-to-Ao ≥ 0.75, enable objective CT-based differentiation of normal and abnormal conditions in dogs, independent of breed or size. While supporting non-invasive pathology detection, these criteria complement—but do not replace—histopathology for definitive diagnosis. This work advances veterinary imaging by filling a critical gap in standardized HLN evaluation, paving the way for improved clinical outcomes in canine abdominal disease management.

## DATA AVAILABILITY

The supplementary data can be made available from the corresponding author upon request.

## AUTHOR’S CONTRIBUTIONS

NC, YH, PC, UH, and PW: Conception and design of the study, data validation and statistical analysis, and drafted and edited the manuscript. YH, PC, UH, and PW: Sample and data collection, performed the experiments, and analyzed the data. CT and SS: Supervised the research. All authors have read and approved the final version of the manuscript.
